# The effectiveness of celebrities in conservation marketing

**DOI:** 10.1371/journal.pone.0180027

**Published:** 2017-07-07

**Authors:** Elizabeth Duthie, Diogo Veríssimo, Aidan Keane, Andrew T. Knight

**Affiliations:** 1 Department of Life Sciences, Imperial College, London, Silwood Park Campus, Ascot, Berkshire, United Kingdom; 2 Andrew Young School of Policy Studies, Department of Economics, Georgia State University, Atlanta, United States of America; 3 School of Geosciences, University of Edinburgh, Edinburgh, United Kingdom; 4 Centre of Excellence in Environmental Decisions, The University of Queensland, St. Lucia Queensland, Australia; 5 Department of Botany, Nelson Mandela Metropolitan University, Port Elizabeth, South Africa; 6 The Silwood Group, Silwood Park Campus, Ascot, Berkshire, United Kingdom; UNITED KINGDOM

## Abstract

Celebrities are frequently used in conservation marketing as a tool to raise awareness, generate funding and effect behaviour change. The importance of evaluating effectiveness is widely recognised in both marketing and conservation but, to date, little research into the effectiveness of celebrity endorsement as a tool for conservation marketing has been published. Using a combination of interviews and an online choice survey instrument, we investigated the extent to which a sample of UK-based conservation organisations, and other charities, evaluate their own usage of celebrity endorsement, and then carried out an experimental evaluation of a hypothetical marketing campaign. This experiment compared participants' willingness-to-engage (WTE) with, and recall of, a conservation message presented in versions of an advert featuring one of three prominent UK celebrities (David Beckham, Chris Packham or HRH Prince William) or a non-celebrity control treatment (featuring Crawford Allan, a director of TRAFFIC USA). We find that the organisations we interviewed did not routinely evaluate their marketing campaigns featuring celebrities. Furthermore, our experiment provides evidence that celebrity endorsement can produce both positive and negative effects. Participants were more willing to engage when presented with an advert featuring one of the three celebrities than the non-celebrity control, and WTE varied according to the characteristics of the celebrity and the respondent. However, celebrities were less effective at generating campaign message recall than non-celebrities. These findings suggest that celebrity endorsement should be used carefully. Further work is required to fully understand the role celebrity endorsers can play in conservation but, drawing on best practice from the field of marketing, this study introduces an approach to evaluation which could be applied more widely to improve the effectiveness of conservation marketing.

## Introduction

It is increasingly acknowledged that effective conservation is ultimately dependent on influencing human attitudes and behavior [1,2]. As with most businesses, conservation non-governmental organizations (NGOs) are dependent on marketing their products and services to the broader populous to effectively and efficiently realize their goals. The field of conservation marketing is increasingly being recognised as an important component in the conservation communities’ toolkit, demonstrated recently by the formation of the Society for Conservation Biology’s Conservation Marketing and Engagement Working Group (ConsMark) [3]. A key strategy in marketing conservation has been the use of celebrities to promote environmental and conservation NGOs and their work [4–7]. For example, His Royal Highness The Duke of Cambridge was instrumental in the creation of the *United For Wildlife* partnership [8], whilst American actors Harrison Ford and Leonardo DiCaprio are, respectively, the Vice-Chair for Conservation Internationals’ Board of Directors [9] and donor, campaigner and board member of the World Wildlife Fund (WWF-US) [10]. Leonardo Dicaprio’s acceptance speech at the 2016 Academy Awards focused on climate change, resulting in a substantially higher volume of news articles, social media posts and information searches than either the 2015 Conference of the Parties or Earth Day [11].

The marketing industry has developed a range of theories and selection techniques over the last six decades to identify the most effective celebrity attributes for specific types of campaigns [12]. These include the source credibility model, which posits that celebrities considered knowledgeable and trustworthy positively impact on the effectiveness of a marketing campaign [13,14], and the product match-up hypothesis, which posits the effectiveness of an advertising campaign increases when there is a clear and identifiable link between the celebrity and a product or service [15,16]. However, despite the wide use of celebrities for marketing products, the empirical literature is equivocal on the effectiveness of celebrity endorsement. Pringle [17] argues that celebrities act as indicators of quality, providing a brand with increased publicity and exposure as well as access to a celebrity’s audience. In addition, a brand is linked by association to desirable qualities that consumers believe a celebrity to possess. Consequently celebrity endorsement may generate and retain attention, increase product recall rates in overly cluttered markets [18,19], and can be a powerful predictor of an intention-to-purchase products or services [20]. However, some studies have found no significant positive effect of celebrity endorsement (e.g., [21,22]) and that celebrity endorsement does not guarantee mass media coverage [23]. If not carefully managed, celebrities, as with flagship species used to promote conservation campaigns [24], may become over-exposed by being associated with too many campaigns, thus diluting their effectiveness [25]. It may even more fundamentally be that celebrity endorsement targeting the broader public is misplaced, as most donations arise from personal relationships, as opposed to celebrity influence [7].

Given the rapid pace of environmental destruction, the limit of available funding [26] and media coverage potentially available to support conservation [27], and donors’ desire to see a high return-on-investment, it is essential that conservation organizations understand the effectiveness of their marketing. This requires evaluation of their marketing initiatives to understand how consumers perceive their product or service, where it sits within the market place, and consequently, what refinements can positively alter consumers’ choices [28]. The marketing industry undertakes extensive testing to ensure that products and services are recognized and viewed positively by consumers, but the effectiveness of marketing conducted by conservation organizations (NGOs) is rarely assessed (although see [28]; [7]). This absence of evaluation is surprising for three reasons. Firstly, the conservation sector relies heavily on celebrity endorsement to increase brand credibility, provide access to new audiences, provide opportunities to effect behavior change in target markets, provide opportunities to raise awareness (either for an organization, or for a specific issue) and provide opportunities to raise funds, either for organizations’ or for specific programmes [7]. Secondly, marketing campaigns are expensive and funding for them limited, meaning ineffective campaigns waste limited resources. Finally, the conservation sector is increasingly promoting evidence-based practice [29] of which evaluation is an essential component.

This study examines how celebrity endorsement is applied by NGOs in the United Kingdom and tests whether celebrity endorsement influences the public’s willingness-to-engage (WTE) with, and subsequent recall of, conservation organizations’ marketing campaigns. Respondent’s perceptions of, and responses to, conservation campaigns featuring different celebrities were also explored. This research demonstrates the limitations of poorly-designed marketing campaigns driven by celebrity endorsement, and the importance of evaluating the marketing initiatives of conservation organizations.

## Materials and methods

The marketing industry’s standard process for evaluating campaigns prior to release is referred to as *pre-testing* and can be subdivided into *concept testing* (an exploratory technique used for preliminary ideas) and *copy testing* (presenting different versions of the same advertisement and evaluating which variant of the attribute being tested is most effective) [30]. For this study, we used a combination of semi-structured interviews (SSIs) and an online copy-testing experiment consisting of two choice tasks and an accompanying questionnaire survey.

### Semi-structured interviews

We conducted SSIs to examine how conservation practitioners perceive and use celebrities in their campaigns. Interview guides were developed based on prior discussions with NGO staff and the literature on celebrity endorsement best practice and covered perceptions of benefits, methods of evaluating effectiveness and celebrity relationship management. The SSIs were arranged through snowball sampling with marketing personnel from 10 conservation-focused NGOs within the United Kingdom [31]. Additionally personnel from two large child protection charities with dedicated celebrity management teams were interviewed, thereby providing an alternative perspective and operational strategy. Where possible, face-to-face meetings were arranged, if this was unfeasible, the interview was held via telephone or email. All SSIs were conducted between May and June 2014.

### Online copy-testing experiment

Two key measures of effective marketing are an audience’s intention to engage with an advertisement’s message (e.g. the intention to adopt a given behavior such as signing a petition, making a donation or purchasing a product or service) and their subsequent ability to recall the information the advertisement was intended to convey. For the purposes of this study, we defined a respondent’s willingness-to-engage (WTE) as the probability they would click on a link contained in an online advertisement. Recall was defined as the probability that a respondent could correctly describe the message of an advertisement. Our copy-testing experiment was designed to investigate how these measures of effectiveness were affected by celebrity endorsement in a conservation setting.

Based on focus-group discussions (see Supporting Information), four advertisements (treatments) were developed in accordance with advertising pre-testing procedure [32,33], each featuring a different male celebrity (three celebrities who have previously endorsed conservation or environmental causes–David Beckham, Chris Packham and Prince William, Duke of Cambridge–and one non-celebrity–Crawford Allan, Senior Director of TRAFFIC). David Beckham is an English footballer who captained the England team for six years and is married to the fashion designer Victoria Beckham. Chris Packham is an English naturalist, author and television presenter, known in the United Kingdom for his frank views on conservation and wildlife, e.g. stating that he believed pandas should be allowed to become extinct [34] and his recent work on bird hunting in Malta [35]. The advertisements were made as similar as possible (within the constraints of the study), with consistent layout, format and presentation (see Supporting Information). Publically available images of the selected individuals were used, coupled with statements each had previously made. Since the consumer behavior and cognitive psychology literatures show a “picture-superiority effect” (that pictures or images are more effective than words in conveying messages) for both memory and judgment, it was considered acceptable for each celebrity to have their own quote [36–38].

The four alternative advertisements formed the basis for our online copy-testing experiment, which comprised two choice tasks and an accompanying questionnaire survey (see Supporting Information). Prior to dissemination, the survey was pilot tested on 17 individuals, aged from 18 to 63, of both genders and with varying degrees of interest in conservation. The feedback was positive, with only minor issues concerning clarification of wording and display issues such as font sizing. Corrections and improvements were made as required. The first choice task examined how respondent WTE and recall was affected by celebrity endorsement (compared with the non-celebrity control) and how these effects differed according to respondent characteristics. Each respondent was shown one of the four advertisements randomly selected by Qualtrics, an online data collection and analysis software package [39] and asked to state whether they would click on the associated link to find out more (i.e. whether or not they were willing-to-engage). Subsequently, a set of questions was posed to respondents to gather data on their: 1) attitude towards the person; 2) awareness of the involvement of that person with a specified campaign; and 3) belief in the person’s reasons for appearing in the advertisement. At the end of this section respondents were asked whether they could remember what the campaign was about to measure their recall.

The second choice task sought to understand which of the four advertisements most appealed to respondents. The four advertisements were presented side-by-side for comparison and respondents were asked to state whether or not they agreed with a series of statements about the advertisements (e.g. “His photo caught my eye”). Next, respondents were instructed to state which of the advertisements would make them most likely to click on the accompanying link, and then asked to select one of several statements describing why they preferred the celebrity (e.g. “I like him more than the others”). Finally respondents were presented with a series of questions about their personal characteristics (e.g. gender, age, donation practices).

The survey was disseminated through conservation organization volunteer mailing lists, social networks (Twitter and Facebook) and social mailing lists such as book groups. Respondents were asked to forward the survey on to three of their friends upon completion in order capture a larger sample. In total 535 people were approached to participate between 17th July and 9th August 2014 and 379 responded (71%). Of these, 16 did not provide a preference in the second choice task reducing the final sample to 363 respondents for that section.

### Ethics

The project was designed in full accordance with the Marketing Research Association’s (MRA) Code of Marketing Research Standards [40] and was approved under the ethics review process of the MSc in Conservation Science at Imperial College London. Free, voluntary, prior informed verbal consent was obtained for all focus group, survey and SSI participants. Verbal consent was deemed sufficient, and authorised by the aforementioned ethics review process, given the nature and subject matter of the focus groups and surveys. SSI participants were contacted individually and their decision to participate was considered verbal consent, similarly authorised by the ethics review process. No minors were involved in this study. Survey respondent anonymity was ensured by not collecting any personally identifiable information (PII), such as location or email address. Focus group and SSI responses were coded to ensure anonymity. Due care was taken to ensure both the source materials and questions were presented impartially, the personalities were not misrepresented, the statement was a direct quote and the images used were publically available and credited appropriately. All participants were thanked upon completion, and provided with contact details for securing further information if required. Feedback on key findings was provided to those who participated in the SSIs and to any other participants who requested it. SSI responses have been kept anonymous at the request of the interviewees.

### Analysis

Data from the copy-testing experiment were analyzed using R version 3.2.1 [41]. Across the sample, 72 individuals failed to answer one or more of the survey questions used to generate predictor variables (see Supporting Information). Rather than remove the responses associated with these individuals–which can bias results if patterns of non-response are non-random–we used the multiple imputation procedure implemented in the mi package [42] to replace the missing data with values sampled from their predictive distribution based on the remainder of the observed data. In keeping with best practice, we performed 10 such imputation draws and averaged the results of analyses conducted separately on each draw [42].

We modeled the effects of celebrity endorsement on respondents’ WTE and recall using Bayesian cumulative logit models to accommodate the ordinal nature of these variables. Predictor variables were included in the model to represent the respondents’ knowledge of the celebrity, their explanation for their choice, beliefs about the celebrity’s motivation for appearing in the advertisement and their individual characteristics (see Supporting Information). We used weakly informative Normal priors (mean = 0, standard deviation = 5) on the parameters representing cut-off points and weakly informative Cauchy priors (location = 0, scale = 2.5) on the beta parameters [43].

We modeled respondents’ choices when presented with all four versions of the advertisements using a Bayesian multinomial logit model with both alternative specific and individual specific predictor variables (see Supporting Information). Alternative specific predictor variables (respondents’ attitudes towards each of the advertisements) were modeled using common parameters while individual specific variables (respondents’ explanations for their choice and their individual characteristics) were modeled with alternative specific parameters. We used weakly informative Cauchy priors (location = 0, scale = 2.5) on both types of predictor variable [43].

The models were fitted using Stan version 2.7.0 [44]. Four Markov Chain Monte Carlo (MCMC) chains were run in parallel for each model for 2,500 iterations, with the first 500 iterations discarded as burn-in. Convergence was assessed using trace plots and Gelman-Rubin statistics, with values < = 1.01 taken to indicate adequate convergence had been obtained [45]. We also checked the effective sample size and Monte Carlo Standard Error (MCSE) of each parameter as indicators of the adequacy of MCMC chain length [45]. We present results from the fitted models as odds ratios (OR) accompanied by 95% credible intervals (95% CI), where an OR < 1 indicates a negative effect and an OR > 1 indicates a positive effect.

## Results

### Use and evaluation of celebrities by organizations

Of the U.K. conservation organizations, all used celebrities to a greater or lesser extent in their marketing and/or fund-raising work and all considered celebrities valuable. Celebrity use differed across organizations, some seeking ‘credibility’ by association, whilst others sought increased social media and press engagement. Accessing new market sectors often involved celebrities appearing at fund-raising events (e.g., as after dinner speakers) to attract high net-worth individuals in influential careers (‘elites’) with whom the organization planned to build a relationship.

All organizations monitored their celebrity-based marketing using web analytics, column inches or social media engagement. Only one of the 10 conservation organizations, and both humanitarian organizations, applied formal evaluation procedures. No evaluation of the effectiveness of specific celebrity attributes was conducted by any organization.

All conservation organizations lacked a strategy for developing and maintaining celebrity partnerships. Most had no formal celebrity engagement strategy, and interactions with celebrities were usually opportunistic and often conducted in an *ad hoc* manner.

Every organization stated they had not paid a celebrity to appear or act as a spokesperson, particularly if the celebrity was a patron or trustee. Some organizations would cover celebrities’ costs incurred by involvement (e.g., travel costs).

### The effectiveness of celebrity endorsement

#### Do celebrities ensure higher WTE?

**Fig 1:**
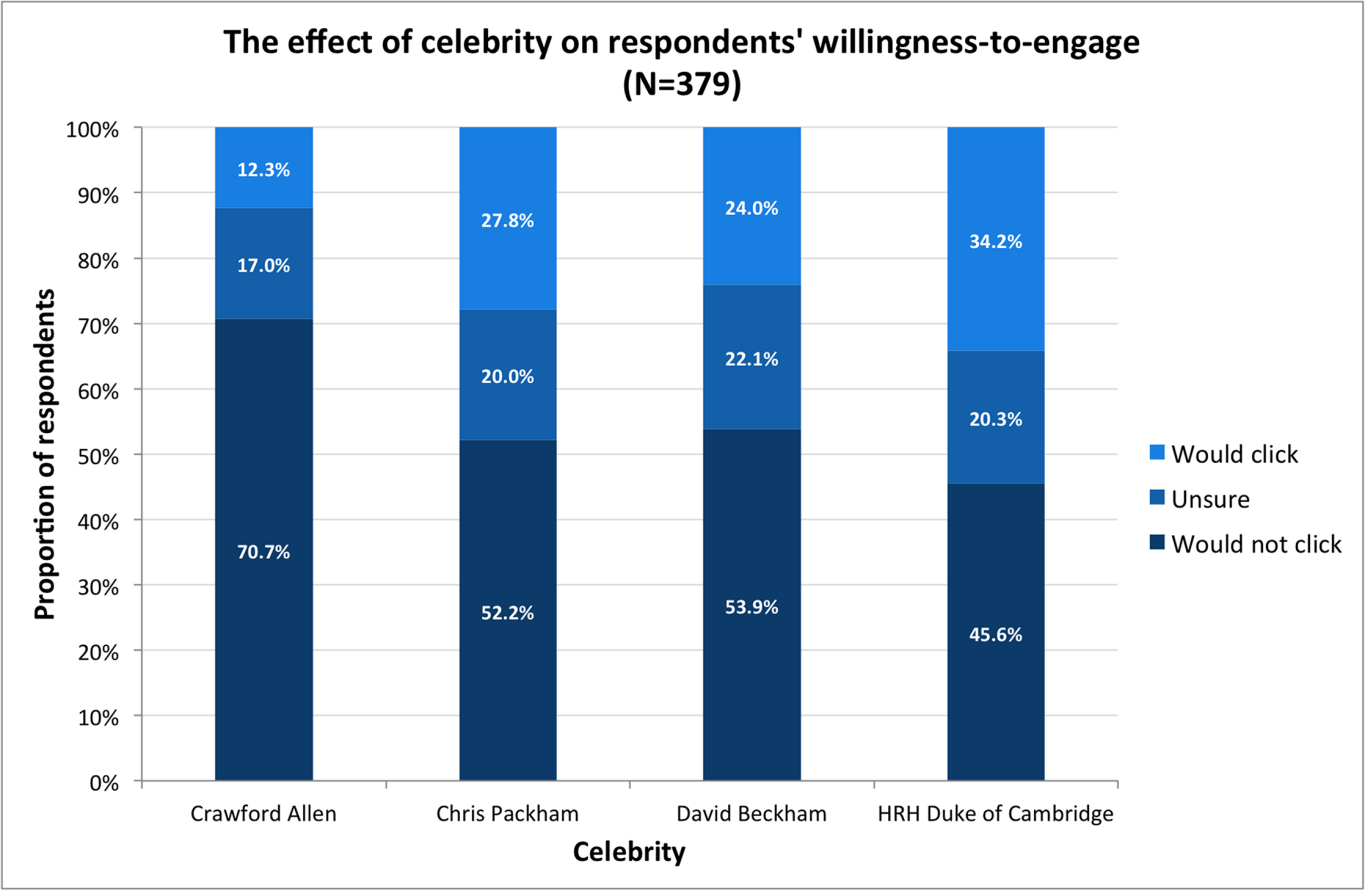
The effect of celebrity on survey respondents’ willingness-to-engage with the advertisement shown to them.

**Fig 2:**
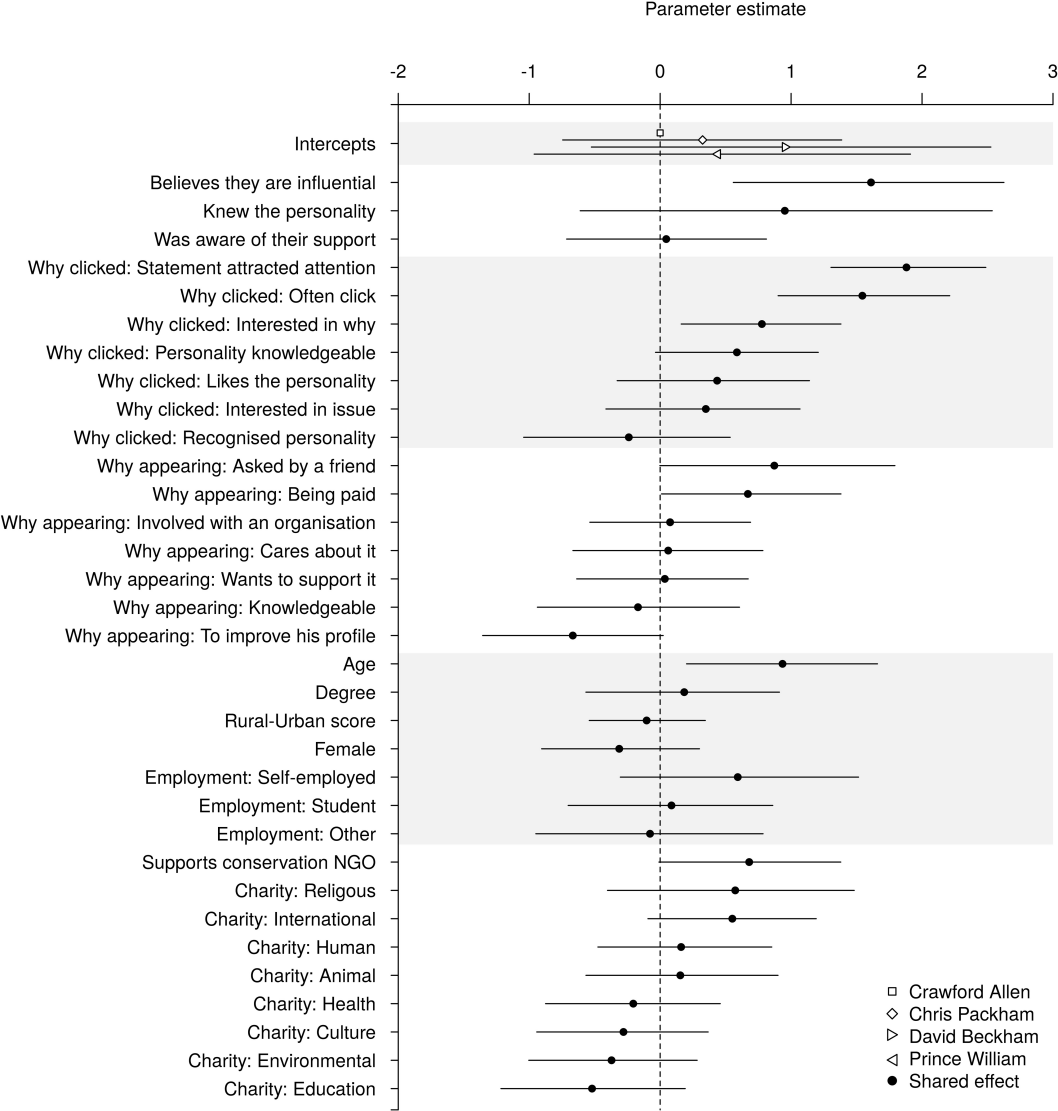
The effects of the celebrity featured and the respondents’ beliefs about them, reasons for clicking, beliefs about the celebrities’ reasons for appearing and their own demographic characteristics on their willingness-to-engage with a single, randomly selected advertisement. Positive values indicate that the variable increases WTE, while negative values indicate that it decreases WTE. Mean estimates (points) are accompanied by 95% credible intervals, calculated as highest posterior density intervals (lines). Grey shading identifies groups of conceptually-related predictor variables. Within these groups predictors are presented in order from most positive to most negative effect to facilitate visual comparisons.

Respondents displayed higher WTE with a celebrity compared to the control (Fig 1). The effect of celebrity was linked to the respondents’ beliefs about the celebrities featured in the advertisements. Respondents who believed that other people would be influenced by the support of the celebrity displayed substantially higher WTE than those who did not (Odds ratio, OR: 5.00; 95% CI: 1.75, 13.81; Fig 2). Respondents reporting a celebrity’s statement caught their attention (OR: 6.56; 95% CI: 3.69, 12.02) or that they often engage with promotional images (OR: 4.68; 95% CI: 2.46, 9.12) displayed higher WTE. Smaller positive effects occurred where respondents displayed interest in why a celebrity supported a campaign (OR: 2.18; 95% CI: 1.17, 3.98) or if they believed the celebrity was knowledgeable about it (OR: 1.80; 95% CI: 0.97, 3.35).

Respondents’ beliefs about why a celebrity was appearing in an advertisement influenced willingness-to-engage (Fig 2). Those who believed a celebrity appeared because a friend had asked (OR: 2.39; 95% CI: 1.00, 6.01) or because they were being paid (OR: 1.95; 95% CI: 1.01, 3.98) were more likely to engage. Conversely, those believing a celebrity appeared to improve their own profile were less likely to (OR: 0.51; 95% CI: 0.26, 1.02).

Older respondents, those who supported one or more conservation NGOs and those who donated to charities focused on international issues, were also more willing-to-engage (Fig 2). In combination, scenario-based predictions from our fitted models suggest an individual’s characteristics can substantially affect their WTE. For example, the fitted model predicts that 43.6% (CI: 22.5%–64.4%) of females, aged 55–64, with a university degree and living in the countryside would engage with Prince William, Duke of Cambridge, compared with only 15.3% (CI: 4.9%–27.0%) of males, aged 16–24, with no university degree and living in a major city (in both cases considering scenarios where the individuals are employed, do not support any conservation NGOs and do not donate to any charities).

#### Do celebrities increase message recall?

**Fig 3:**
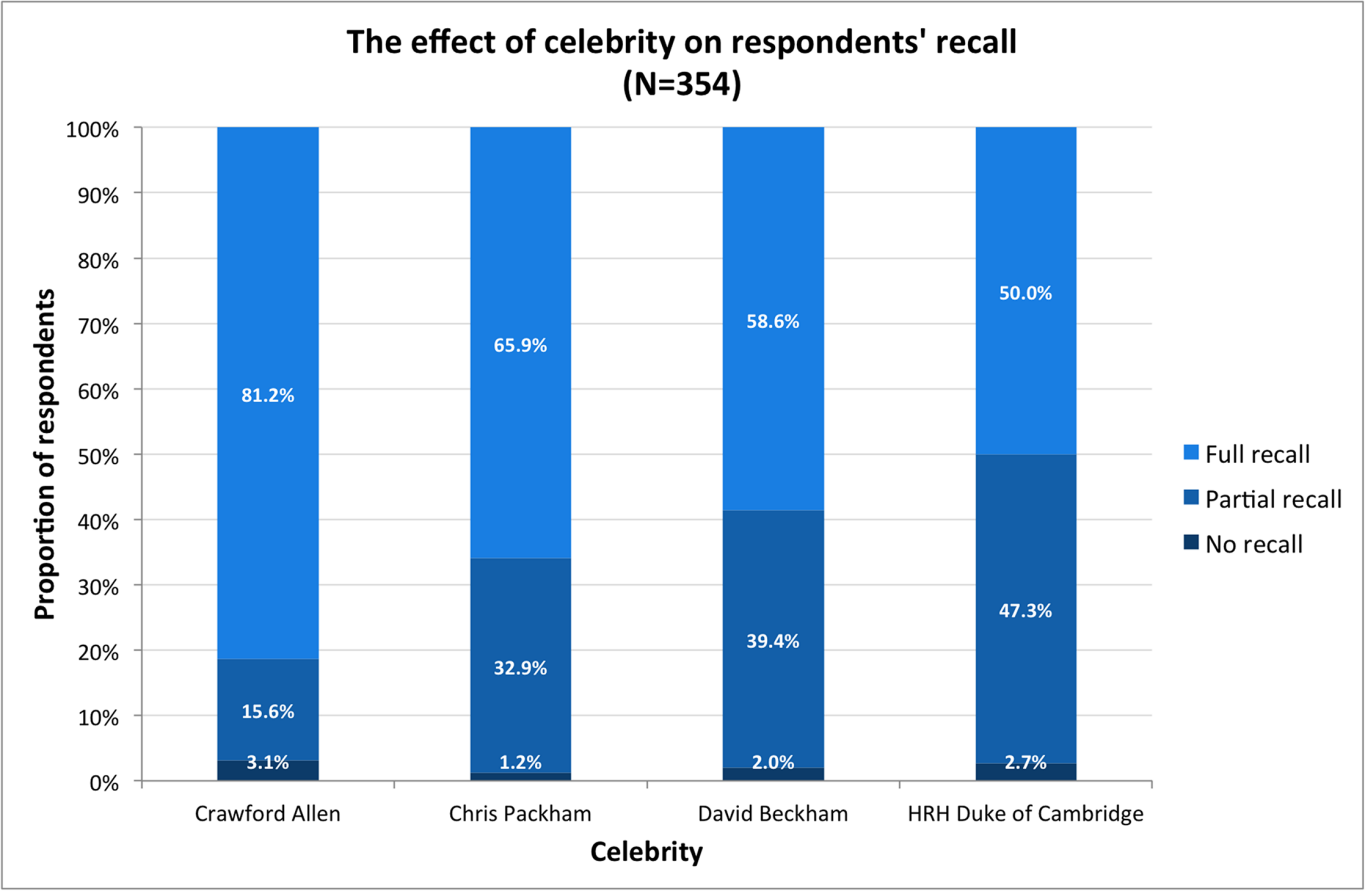
The effect of celebrity on survey respondents’ ability to recall campaign message of the advertisement shown to them.

**Fig 4:**
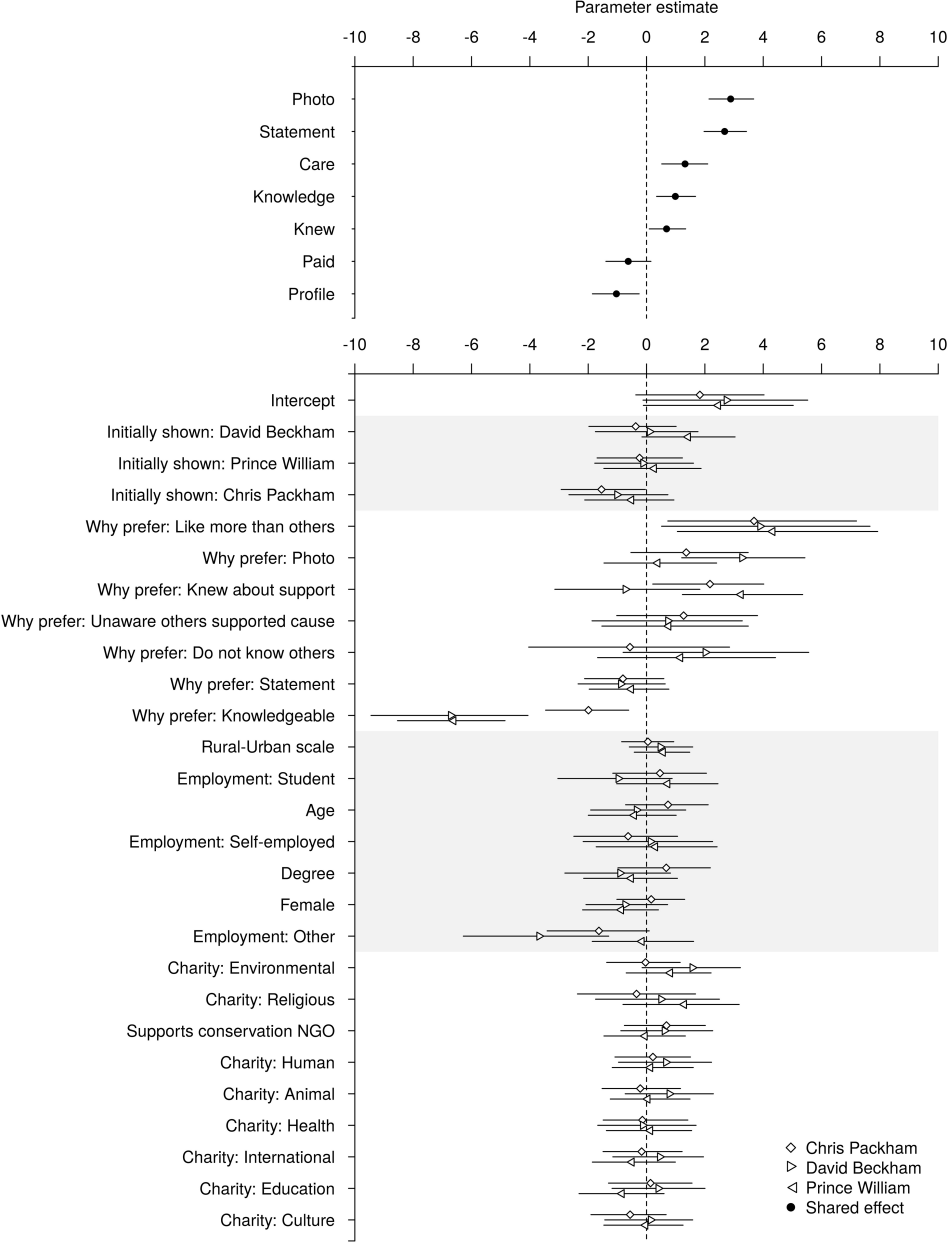
The effects of advertisement-specific characteristics (Fig 4A). **The celebrity featured and individual-specific respondent characteristics (Fig 4B) on the advertisement chosen when all four were presented alongside one another.** The parameter estimates are presented on the scale of the linear predictor. Positive values indicate that the variable increases the probability that the celebrity is chosen relative to the baseline category (Crawford Allan), while negative values indicate that it decreases the probability that he is chosen. Mean estimates (points) are accompanied by 95% credible intervals, calculated as highest posterior density intervals (lines). Grey shading is used to highlight groups of conceptually-related predictor variables. Within these groups, the sets of parameters associated with each predictor variable are ordered by the set mean from most positive to most negative effect to facilitate visual comparisons.

Respondents were less likely to recall an advertisement message if it featured a celebrity than if it featured the control (Fig 3). Aside from the specific celebrity featured in the advertisements, few other variables predicted recall well. Recall was somewhat lower amongst older (OR: 0.44; 95% CI: 0.23, 0.84) and female (OR: 0.6; 95% CI: 0.34, 1.08) respondents but higher amongst those who reported donating to religious charities (OR: 2.54; 95% CI: 0.98, 6.65) but no other predictors produced clear effects (Fig 4).

#### Why do respondents choose one celebrity over another?

When presented with all four advertisements and asked to choose which they preferred, the advertisement featuring Chris Packham was the most popular (chosen by 36.9% of respondents), followed by Prince William, Duke of Cambridge (34.2%), David Beckham (16.8%) and Crawford Allan (12.1%).

Respondents' choice was predicted by advertisement characteristics, stated preference for a specific celebrity, and demographic characteristics. The most important advertisement characteristics were the photograph, then the statement. Respondents were more likely to choose a celebrity when they reported that "His photograph caught my eye" (OR: 17.88; 95% CI: 8.52, 39.34) or "His statement made me want to find out more" (OR: 14.55; 95% CI: 7.19, 30.73; Fig 4A).

Respondents were also more likely to choose a celebrity if they believed he cared about (OR: 3.75; 95% CI: 1.68, 8.14), was knowledgeable about (OR: 2.68; 95% CI: 1.41, 5.36), or was known to support (OR: 1.99; 95% CI: 1.1, 3.82) a specified issue. Celebrities thought to be using an issue to improve or raise his own profile (OR: 0.36; 95% CI: 0.16, 0.78) or might be being paid for their involvement (OR: 0.53; 95% CI: 0.25, 1.16) were less likely to be preferred.

Respondents who explained their choice by stating “I like him more than the others” were much more likely to prefer Prince William, Duke of Cambridge (OR: 75.26; 95% CI: 2.88, 2762.31), followed by David Beckham (OR: 49.08; 95% CI: 1.67, 2120.99), Chris Packham (OR: 39.83; 95% CI: 2.08, 1335.76) and Crawford Allan (Fig 4B). Respondents who explained their choice by stating “His photo caught my eye” were more likely to choose David Beckham (OR: 26.43; 95% CI: 3.34, 227.91), while those who stated “I already knew about his support for this” most often chose Prince William, Duke of Cambridge (OR: 25.41; 95% CI: 3.42, 211.03) or Chris Packham (OR: 8.80; 95% CI: 1.24, 55.58). Conversely, those who selected “I believe he is the most knowledgeable about this issue” were most likely to choose Crawford Allan, followed by Chris Packham (OR: 0.14; 95% CI: 0.03, 0.54) and were very unlikely to choose Prince William, Duke of Cambridge (OR: 0.00; 95% CI: 0.00, 0.02) or David Beckham (OR: 0.00; 95% CI: 0.00, 0.01).

## Discussion

Marketing, having existed in some form since humans started trading, has a well-established body of theory, [46,47], and is a key activity in business practice [48]. It has evolved from concentrating on the product to the current focus on relationship marketing; the constant connection and interplay between the consumer, product and brand [49]. Consequently many NGOs are increasingly using new marketing thinking and practice to guide their strategies and communications [50]. Marketing strategies provide the reference point for all decisions an organization makes about its marketing concepts and outputs and are critically important for ensuring resources are used effectively and brand reputation is maintained and developed [51]. The conservation NGOs in this study, and possibly those more generally, lack a dedicated celebrity endorsement strategy. This is problematic, given the on-going and extensive reliance upon celebrity endorsement and its perceived effectiveness. Furthermore, a lack of formal celebrity endorsement strategy results in a failure to evaluate celebrity campaigns and therefore understand the ways in which to maximize the return-on-investment (ROI). This study found that whilst celebrities can be beneficial in eliciting positive WTE behavior, they can have a negative effect on message recall, and the choice of celebrity can play a critical role in the effectiveness of a campaign.

The role of evaluation and evidence-based analysis is considered a priority within the marketing industry [52,53], just as it is within the conservation community [29,54]. However, while conservation organizations commonly use celebrities to market their campaigns, our results suggest that they rarely pre-test or evaluate (i.e., employ evidence for) their effectiveness. Our findings suggest that celebrity endorsement is indeed able to generate higher levels of WTE amongst the public, corroborating the results of previous studies [55,56]. However, they also challenge the idea that celebrity endorsement is always beneficial, regardless of application–a view that was commonly expressed in our interviews with representatives of conservation organizations–by showing that the public’s WTE with celebrities in conservation campaigns is nuanced, and driven by multiple factors, with no one approach or technique being universally effective [6].

Celebrities therefore may not always be the most effective choice for a marketing campaign and appropriate evaluation is essential. For example, in accordance with the principles of the Source Credibility Model [57,13], we found that celebrities who were considered to be knowledgeable about an issue generated significantly higher levels of WTE. As Till & Busler [22] argue, this suggests that celebrity endorsers should ideally be seen to possess expertise about the product or topic they are promoting. Interestingly, whilst Chris Packham was viewed as being highly knowledgeable about the illegal wildlife trade, he had never officially spoken about the issue prior to the data collection period. Thus, his perceived expertise most likely derives from his role as a nature documentary presenter and campaigner on various wildlife and conservation related issues (cf. [57,58]). By contrast, Crawford Allan was viewed as the most knowledgeable (understandably, given his role as Senior Director for Wildlife Crime at Traffic), but this expertise alone was not enough to generate higher levels of engagement.

It is also important that evaluations of the effectiveness of celebrity endorsement take great care when selecting their measures of success. Crucially, while we found that celebrity endorsement generates higher WTE, it also led to lower recall of the issues being communicated [59]. There are several factors that may have contributed to this finding. One possibility is that if a celebrity’s support for a broader issue is well established, that existing association might complicate attempts to communicate a more specific message, resulting in an ineffective campaign. For example, Prince William, Duke of Cambridge has become well known for his involvement in wildlife conservation (despite the British Royal Family’s ongoing interest and participation in blood sports [60]) and particularly his role in forming *United for Wildlife*, a collection of seven conservation organizations combatting the illegal trade in wildlife. Given the extensive press exposure *United for Wildlife* has received, particularly in the United Kingdom, under the product match-up hypothesis [15,16] it would have been reasonable to expect that he would elicit higher levels of recall from respondents in the survey. In fact, while levels of full recall were low, he generated reasonable levels of incomplete recall, suggesting that respondents may only have been able to recall that Prince William was associated with wildlife conservation in general. They were not able to remember the exact nature of the issue he was endorsing. A second possibility relates to the directed nature of the survey, in which respondents were asked to look at the advertisement and answer questions accordingly. This is not reflective of the contextual nature of marketing, with respondents instructed to examine an advertisement they might otherwise ignore. Additionally, given the unfamiliarity of Crawford Allan for the majority of respondents, additional cognitive processing would have been necessary, thus resulting in higher levels of recall [59].

While promoting more widespread evaluation of celebrity endorsement–and conservation marketing more generally–we recognize that conservation organizations face multiple barriers that might hinder its adoption. In addition to pressure on resources (financial, staff time and arguably staff capacity) within conservation NGOs, it is understandable that organizations do not want to risk wasting a celebrity endorsement opportunity. The majority of celebrity endorsement in conservation marketing is reliant on goodwill and personal connections. With little formal guidance available for selecting an endorser, let alone a specific marketing technique for use, conservation organizations are left grasping at any celebrity endorsement opportunity available. As shown by this study, this does not necessarily translate into effective marketing, and in some cases could potentially be detrimental to an issue or organization. Furthermore, conservation organizations are rarely approached by celebrities seeking partnership opportunities. Instead, organizations draw-up a ‘wish list’ of celebrities they hope to engage with, and successively work down the list until they find ones who are willing and available. Whilst it is understandable that for many organizations this is the only process available to them to secure celebrity endorsement, it runs a substantial risk of identifying a celebrity who exerts little, or worse, negative influence, particularly given the body of literature reinforcing the importance of celebrity endorser and brand congruence.

It is vital to ensure campaigns are as effective as possible, which is determined by factors including the public perception of the brand and the celebrity endorsement itself. Greater research is required to ensure celebrity endorsement is used in the most effective way, for example to avoid the perception that their involvement is undermining or belittling the issue [61]. Future work should aim to understand the celebrity attributes that are most effective, and the demographic groups they are most effective on. Understanding the role celebrities play in communicating conservation ideas and issues at both global and local levels is critical for ensuring celebrity endorsers are used strategically and appropriately. Understanding the effect celebrity endorsement has on decision- and policy-makers will allow the conservation community to maximize the return-on-investment of their marketing campaigns. It is also important to understand if, and in what contexts, charismatic species of fauna and flora, or other elements of nature, are more effective than human celebrities in communicating conservation messages to the public, positively changing behavior and raising funds.

## Conclusion

Celebrity endorsement is used to market conservation projects and programmes, however the reasons conservation NGOs adopt this technique is rarely explored. Furthermore their effectiveness is rarely investigated.

Celebrity endorsement is not unequivocally effective [12], and there is increasing philanthropic fatigue from the public [62]. Therefore a key recommendation, based on the marketing literature, and the findings from this study, are for conservation organizations to focus on building brand awareness and equity. Not only will increased brand presence in the market increase public engagement, but increased brand equity (the assets such as name recognition and perceived quality that are connected to a brand and give a product or service its value [63]) will serve as a useful tool in securing celebrity endorsement. Given the lack of marketing research currently conducted by conservation organizations, this is an area where the research community could make a substantial and useful contribution to conservation organizations. Researchers can bypass the risk to reputation and celebrity endorser relationships that conservation organizations may carry if they undertake the research themselves. Whilst celebrities can prove more effective in generating positive WTE behaviour, they might not be as effective at generating full campaign message recall and factors such as credibility, likeability and a connection between the campaign and endorser are critical. This study shows the role testing and evaluation can play in ensuring the maximum impact of celebrity endorsers and how they can be harnessed to not only raise funds for conservation, but also to raise awareness and effect behaviour change.

## Supporting information

S1 Appendix Additional methods used in this study. (DOCX)

S2 Appendix The advertisements used in the online survey. Images, provided by Stephanie O’Donnell (CC BY 4.0), are similar but not identical to the original images and therefore are for illustrative purposes only.
(DOCX)

S3 Appendix The full online survey. (DOCX)

S4 Appendix Predictors used in models of respondents’ willingness-to-engage with (W) and recall (R) of adverts presented singly and choices (C) between four alternative adverts presented side by side. (DOCX)

S1 Data Copy test. (CSV)

S2 Data Choice experiment. (CSV)
