# Supplementary material for: The effectiveness of celebrities in conservation marketing
Source: PLoS One. 2017 Jul 7;12(7):e0180027. doi: 10.1371/journal.pone.0180027 (PMC5501471; doi:10.1371/journal.pone.0180027)
Supplement: S1 Appendix — (DOCX) [file pone.0180027.s001.docx]

**Appendix S1:**

**Further information about the methods used in the study**

*Focus group discussions*

Focus groups were conducted to support the design and structure of our online choice task, with participants asked to discuss their perceptions of, and attitudes towards, celebrities in the campaigns of conservation NGOs. Five focus groups were held in May and June 2014, with three of the groups made up of individuals with no strong interest in, or ties to, any conservation organisation. The remaining two groups consisted of individuals who self-identified as having an interest in conservation, for example as volunteers for a conservation organization. Where possible, the participants in each group fell within the same age group and/or socio-economic division, to ensure comfort, freedom of expression and a free-flowing conversation.

*Focus group questions*

**Introduction:**

Thank you so much for agreeing to be part of this discussion. My name is Lizzie Duthie, and as part of my Masters in Conservation Science at Imperial College, I am investigating the impact celebrities have in conservation marketing. This discussion will help me to learn more about your opinions on conservation marketing and the role celebrities do or don’t play.

I need to let you know that this discussion is being tape-recorded, and notes are being taken by ____________________. We are recording the discussion so I can ensure I have a faithful transcript of the session, however your comments are confidential, and no one but myself will listen to the recording. Any comments written up, as part of the research, will remain anonymous.

I am just going to go over the ground rules briefly. I want to hear everything you say, so I’ll ask you to speak one at a time, and loud enough for the recorder. I’m going to ask you a few questions, to which there are no ‘right’ or ‘wrong’ answers – anything you want to say is ok, and I want to hear what you like and don’t like – everything you say is useful, and please feel free to speak up whether you agree or disagree. I would also like everyone to participate and so I may call on you if I haven’t heard from you in a while.

1.) Let’s begin with a quick exercise. Please can you make a mental note of the advert that most appeals to you, and then we will go round in a circle and everyone can take a turn to briefly introduce themselves and explain why they chose that advert.

2.) Were there any adverts that you found unclear or didn’t understand?

3.) Does the advert you chose actually make you want to donate/sign up/learn more? If not, why not?

4.) Can you think of any changes that could be made to the advert to make it more likely to make you take action?

6.) Celebrities: I am particularly interested in the role celebrities can play in conservation marketing, and so I wanted to spend a little time understanding some of the connections between celebrities and conservation.

So can you please write down the names of five celebrities you would expect to front a conservation campaign (of any kind or type). We will then go round in a circle and explain briefly our reasons for choosing them.

7.) So what qualities in particular do you think are necessary for these celebrities to have?

8.) And what sort of qualities would make a celebrity seem like a bad choice?

9.) What would be the single most important factor for you accepting and believing a celebrities involvement and endorsement of a campaign or issue?

10.) And what would be the single most off-putting factor that would make you

11.) What do you think motivates a celebrity to choose to represent a conservation issue or present a campaign?

12.) Do you think knowing the reason behind the celebrities involvement would make you more or less likely to believe them?

13.) That’s brilliant, thank you.

*Summarise what has been said in this section so far and then ask:* How well does that capture what has been said?

14.) Is there anything you would like to add to this discussion that you feel has not already been addressed?

That wraps up our discussion today. Thank you all so much for participating! Your ideas and comments are extremely helpful and will be used to help conservation marketing in the future.
