# Supplementary material for: The effectiveness of celebrities in conservation marketing
Source: PLoS One. 2017 Jul 7;12(7):e0180027. doi: 10.1371/journal.pone.0180027 (PMC5501471; doi:10.1371/journal.pone.0180027)
Supplement: S2 Appendix — Images, provided by Stephanie O’Donnell (CC BY 4.0), are similar but not identical to the original images and therefore are for illustrative purposes only. (DOCX) [file pone.0180027.s002.docx]

**Appendix S2:**

**The four treatments**

**
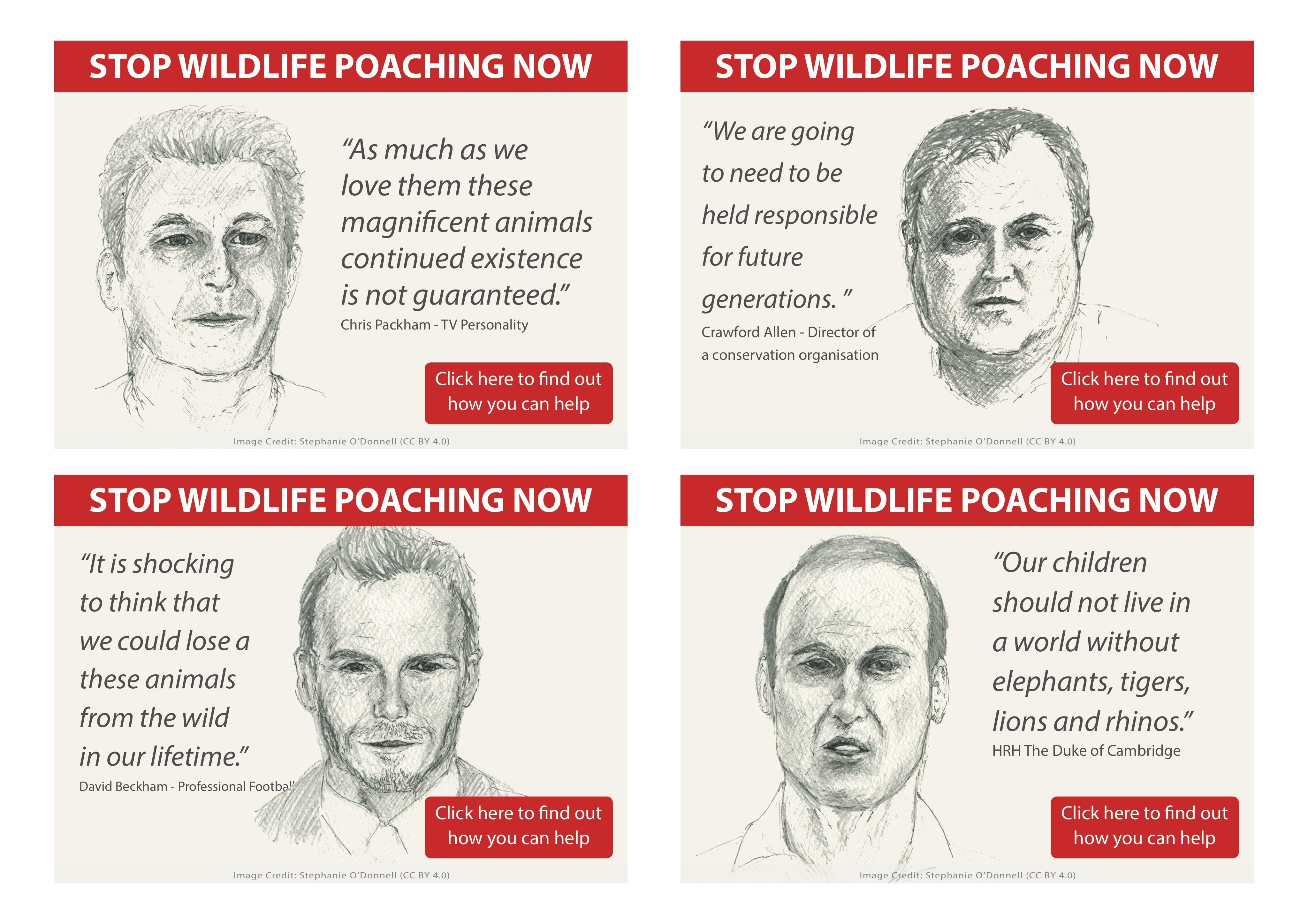
**

**Illustrations by Stephanie O’Donnell (CC BY 4.0). These images are similar but not identical to the original images and therefore are for illustrative purposes only.**
