# Supplementary material for: The effectiveness of celebrities in conservation marketing
Source: PLoS One. 2017 Jul 7;12(7):e0180027. doi: 10.1371/journal.pone.0180027 (PMC5501471; doi:10.1371/journal.pone.0180027)
Supplement: S3 Appendix — (DOCX) [file pone.0180027.s003.docx]

**Appendix S3:**

Online survey

Q1.1 Thank you for agreeing to take part in this short survey - it should only take about 5 minutes to complete.

You are about to see a promotional image for a campaign to stop wildlife poaching. Please take a moment to look at it carefully.

Q1.3 Q1: If you saw this image online, would you click on the link to find out more?

- Yes (1)
- No (2)
- Unsure (3)

Answer If Q2: Why is that?Please indicate where on the spectrum your opinion lies: I like him:I don't like him Is Selected Or Q2: Why is that?Please indicate where on the spectrum your opinion lies: I am interested in this issue:I am not interested in this issue Is Selected Or Q2: Why is that?Please indicate where on the spectrum your opinion lies: I believe he is knowledgeable about it:I don't believe he knows enough about it Is Selected

Q1.4 Q2: Why is that?Please indicate where on the spectrum your opinion lies:

|  | 1 (1) | 2 (2) | 3 (3) | 8 (8) | 10 (10) |
| --- | --- | --- | --- | --- | --- |
| I like him:I don't like him (1) |  |  |  |  |  |
| I am interested in this issue:I am not interested in this issue (2) |  |  |  |  |  |
| I believe he is knowledgeable about it:I don't believe he knows enough about it (3) |  |  |  |  |  |
| His statement caught my attention:His statement has no impact on me (4) |  |  |  |  |  |
| I am interested in why he is supporting it:I don't care that he is supporting it (5) |  |  |  |  |  |
| I often click on promotional images like this:I never click on promotional images like this (6) |  |  |  |  |  |
| I instantly recognised him:I don't know who he is (7) |  |  |  |  |  |

Answer If Q1: If you saw this image online, would you click on the link to find out more? Yes Is Selected Or Q1: If you saw this image online, would you click on the link to find out more? No Is Selected Or Q1: If you saw this image online, would you click on the link to find out more? Unsure Is Selected

Q1.5 Q3: Are there any other reasons for your decision?

Q1.6

Q1.7 Q4: Do you know who he is?

- Yes (1)
- No (2)
- Unsure (3)

Answer If Do you know who he is? Yes Is Selected

Q1.8 Q5: Please tell us who you think he is and what he is known for:

Answer If Do you know who he is? No Is Selected Or Do you know who he is? Unsure Is Selected

Q1.9 David Beckham is a former professional footballer who has played internationally and for the England national team. His charitable work includes UNICEF, Elton John AIDS Foundation and Malaria No More UK in addition to his role as Lead Ambassador for United for Wildlife.   Q5: Knowing this information, does his appearance in this campaign make more sense?

- Yes (1)
- No (2)
- Unsure (3)

Answer If Q5: Please tell us who you think he is and what he is known for: Text Response Is Not Empty Or Q5: Please tell us who you think he is and what he is known for: Text Response Is Empty Or David Beckham is a former professional footballer who has played internationally and for the Engl... Yes Is Selected Or David Beckham is a former professional footballer who has played internationally and for the Engl... No Is Selected Or David Beckham is a former professional footballer who has played internationally and for the Engl... Unsure Is Selected

Q1.10 Q6: Were you aware of his support for this issue prior to seeing this promotional image?

- Yes (1)
- No (2)
- Unsure (3)

Answer If Were you aware of his support for this issue prior to seeing this promotional image? Yes Is Selected

Q1.11 Q7: Does it increase your belief in his commitment to this issue?

- Yes (1)
- No (2)
- Unsure (3)

Answer If Were you aware of his support for this issue prior to seeing this promotional image? No Is Selected Or Were you aware of his support for this issue prior to seeing this promotional image? Unsure Is Selected

Q1.12 Q7: What level of committment do you think he has to this issue?

- Strong (1)
- Moderate (2)
- Neutral (3)
- Slight (4)
- None (5)

Answer If Does his continuing support increase your belief in his committment to this issue? Yes Is Selected Or Does his continuing support increase your belief in his committment to this issue? No Is Selected Or Does his continuing support increase your belief in his committment to this issue? Unsure Is Selected Or What level of committment do you think he has to this issue? Strong Is Selected Or What level of committment do you think he has to this issue? Moderate Is Selected Or What level of committment do you think he has to this issue? Neutral Is Selected Or What level of committment do you think he has to this issue? Slight Is Selected Or What level of committment do you think he has to this issue? None Is Selected

Q1.13 Q8: Do you think that other people would be influenced by his support of the issue?

- Yes (1)
- No (2)
- Unsure (3)

Q1.14 Q9: Why do you believe he is appearing in this campaign?Please tick all that apply

- He is knowledgeable about it (1)
- He wants to support it publically (7)
- He is involved in an organisation or charity that is working on this (2)
- He hopes to improve his public profile (3)
- He cares about it (4)
- He is being paid to appear (5)
- A friend asked him to get involved (8)
- Other (please specify) (6) ____________________

Answer If Why do you believe he is appearing in this campaign? Please tick all that apply q://QID95/SelectedChoicesCount Is Greater Than or Equal to 1

Q1.15 Q10: Can you remember his name? If so, please write it here:

Answer If Q9: Why do you believe he is appearing in this campaign?Please tick all that apply q://QID95/SelectedChoicesCount Is Greater Than or Equal to 1

Q1.16 Q11: Can you remember what the campaign is about? If so, please write it here:

Q2.1 Thank you for agreeing to take part in this short survey - it should only take about 5 minutes to complete.     You are about to see a promotional image for a campaign to stop wildlife poaching. Please take a moment to look at it carefully.

Q2.2

Q2.3 Q1: If you saw this image online, would you click on the link to find out more?

- Yes (1)
- No (2)
- Unsure (3)

Answer If Q1: If you saw this image online, would you click on the link to find out more? Yes Is Selected Or Q1: If you saw this image online, would you click on the link to find out more? No Is Selected Or Q1: If you saw this image online, would you click on the link to find out more? Unsure Is Selected

Q2.4 Q2: Why is that?Please indicate where on the spectrum your opinion lies:

|  | 1 (1) | 2 (2) | 3 (3) | 8 (8) | 10 (10) |
| --- | --- | --- | --- | --- | --- |
| I like him:I don't like him (1) |  |  |  |  |  |
| I am interested in this issue:I am not interested in this issue (2) |  |  |  |  |  |
| I believe he is knowledgeable about it:I don't believe he knows enough about it (3) |  |  |  |  |  |
| His statement caught my attention:His statement has no impact on me (4) |  |  |  |  |  |
| I am interested in why he is supporting it:I don't care that he is supporting it (5) |  |  |  |  |  |
| I often click on promotional images like this:I never click on promotional images like this (6) |  |  |  |  |  |
| I instantly recognised him:I don't know who he is (7) |  |  |  |  |  |

Answer If Q1: If you saw this image online, would you click on the link to find out more? Yes Is Selected Or Q1: If you saw this image online, would you click on the link to find out more? No Is Selected Or Q1: If you saw this image online, would you click on the link to find out more? Unsure Is Selected

Q2.5 Q3: Are there any other reasons for your decision?

Q2.6

Q2.7 Q4: Do you know who he is?

- Yes (1)
- No (2)
- Unsure (3)

Answer If Q4: Do you know who he is? Yes Is Selected

Q2.8 Q5: Please tell us who you think he is and what he is known for:

Answer If Q4: Do you know who he is? No Is Selected Or Q4: Do you know who he is? Unsure Is Selected

Q2.9 Chris Packham is a naturalist, author, nature photographer and television presenter. He is best known for presenting programmes such as The Really Wild Show and Springwatch.    Q5: Knowing this information, does his appearance in this campaign make more sense?

- Yes (1)
- No (2)
- Unsure (3)

Answer If Q5: Please tell us who you think he is and what he is known for: Text Response Is Not Empty Or Q5: Please tell us who you think he is and what he is known for: Text Response Is Empty Or Chris Packham is a naturalist, author, nature photographer and television presenter. He is best... Yes Is Selected Or Chris Packham is a naturalist, author, nature photographer and television presenter. He is best... No Is Selected Or Chris Packham is a naturalist, author, nature photographer and television presenter. He is best... Unsure Is Selected

Q2.10 Q6: Were you aware of his support for this issue prior to seeing this promotional image?

- Yes (1)
- No (2)
- Unsure (3)

Answer If Q6: Were you aware of his support for this issue prior to seeing this promotional image? Yes Is Selected

Q2.11 Q7: Does it increase your belief in his commitment to this issue?

- Yes (1)
- No (2)
- Unsure (3)

Answer If Q6: Were you aware of his support for this issue prior to seeing this promotional image? No Is Selected Or Q6: Were you aware of his support for this issue prior to seeing this promotional image? Unsure Is Selected

Q2.12 Q7: What level of committment do you think he has to this issue?

- Strong (1)
- Moderate (2)
- Neutral (3)
- Slight (4)
- None (5)

Answer If Q7: Does his continuing support increase your belief in his committment to this issue? Yes Is Selected Or Q7: Does his continuing support increase your belief in his committment to this issue? No Is Selected Or Q7: Does his continuing support increase your belief in his committment to this issue? Unsure Is Selected Or Q7: What level of committment do you think he has to this issue? Strong Is Selected Or Q7: What level of committment do you think he has to this issue? Moderate Is Selected Or Q7: What level of committment do you think he has to this issue? Neutral Is Selected Or Q7: What level of committment do you think he has to this issue? Slight Is Selected Or Q7: What level of committment do you think he has to this issue? None Is Selected

Q2.13 Q8: Do you think that other people would be influenced by his support of the issue?

- Yes (1)
- No (2)
- Unsure (3)

Q2.14 Q9: Why do you believe he is appearing in this campaign?Please tick all that apply

- He is knowledgeable about it (1)
- He wants to support it publically (7)
- He is involved in an organisation or charity that is working on this (2)
- He hopes to improve his public profile (3)
- He cares about it (4)
- He is being paid to appear (5)
- A friend asked him to get involved (8)
- Other (please specify) (6) ____________________

Answer If Q9: Why do you believe he is appearing in this campaign? Please tick all that apply q://QID133/SelectedChoicesCount Is Greater Than or Equal to 1

Q2.15 Q10: Can you remember his name? If so, please write it here:

Answer If Q9: Why do you believe he is appearing in this campaign? Please tick all that apply q://QID133/SelectedChoicesCount Is Greater Than or Equal to 1

Q2.16 Q11: Can you remember what the campaign is about? If so, please write it here:

Q3.1 Thank you for agreeing to take part in this short survey - it should only take about 5 minutes to complete.     You are about to see a promotional image for a campaign to stop wildlife poaching. Please take a moment to look at it carefully.

Q3.2

Q3.3 Q1: If you saw this image online, would you click on the link to find out more?

- Yes (1)
- No (2)
- Unsure (3)

Answer If Q1: If you saw this image online, would you click on the link to find out more? Yes Is Selected Or Q1: If you saw this image online, would you click on the link to find out more? No Is Selected Or Q1: If you saw this image online, would you click on the link to find out more? Unsure Is Selected

Q3.4 Q2: Why is that?Please indicate where on the spectrum your opinion lies:

|  | 1 (1) | 2 (2) | 3 (3) | 8 (8) | 10 (10) |
| --- | --- | --- | --- | --- | --- |
| I like him:I don't like him (1) |  |  |  |  |  |
| I am interested in this issue:I am not interested in this issue (2) |  |  |  |  |  |
| I believe he is knowledgeable about it:I don't believe he knows enough about it (3) |  |  |  |  |  |
| His statement caught my attention:His statement has no impact on me (4) |  |  |  |  |  |
| I am interested in why he is supporting it:I don't care that he is supporting it (5) |  |  |  |  |  |
| I often click on promotional images like this:I never click on promotional images like this (6) |  |  |  |  |  |
| I instantly recognised him:I don't know who he is (7) |  |  |  |  |  |

Answer If Q1: If you saw this image online, would you click on the link to find out more? Yes Is Selected Or Q1: If you saw this image online, would you click on the link to find out more? No Is Selected Or Q1: If you saw this image online, would you click on the link to find out more? Unsure Is Selected

Q3.5 Q3: Are there any other reasons for your decision?

Q3.6

Q3.7 Q4: Do you know who he is?

- Yes (1)
- No (2)
- Unsure (3)

Answer If Q4: Do you know who he is? Yes Is Selected

Q3.8 Q5: Please tell us who you think he is and what he is known for:

Answer If Q4: Do you know who he is? No Is Selected Or Q4: Do you know who he is? Unsure Is Selected

Q3.9 Crawford Allen is the Senior Director of TRAFFIC, the world&#39;s largest international wildlife trade monitoring organisation. Traffic is run jointly by WWF and the International Union for the Conservation of Nature (IUCN) and Crawford&#39;s role involves combatting the illegal trade in wildlife.   Q5: Knowing this information, does his appearance in this campaign make more sense?

- Yes (1)
- No (2)
- Unsure (3)

Answer If Q5: Please tell us who you think he is and what he is known for: Text Response Is Not Empty Or Q5: Please tell us who you think he is and what he is known for: Text Response Is Empty Or Crawford Allen is the Senior Director of TRAFFIC, the world's largest international wildlife tr... Yes Is Selected Or Crawford Allen is the Senior Director of TRAFFIC, the world's largest international wildlife tr... No Is Selected Or Crawford Allen is the Senior Director of TRAFFIC, the world's largest international wildlife tr... Unsure Is Selected

Q3.10 Q6: Were you aware of his support for this issue prior to seeing this promotional image?

- Yes (1)
- No (2)
- Unsure (3)

Answer If Q6: Were you aware of his support for this issue prior to seeing this promotional image? Yes Is Selected

Q3.11 Q7: Does it increase your belief in his commitment to this issue?

- Yes (1)
- No (2)
- Unsure (3)

Answer If Q6: Were you aware of his support for this issue prior to seeing this promotional image? No Is Selected Or Q6: Were you aware of his support for this issue prior to seeing this promotional image? Unsure Is Selected

Q3.12 Q7: What level of committment do you think he has to this issue?

- Strong (1)
- Moderate (2)
- Neutral (3)
- Slight (4)
- None (5)

Answer If Q7: Does his continuing support increase your belief in his committment to this issue? Yes Is Selected Or Q7: Does his continuing support increase your belief in his committment to this issue? No Is Selected Or Q7: Does his continuing support increase your belief in his committment to this issue? Unsure Is Selected Or Q7: What level of committment do you think he has to this issue? Strong Is Selected Or Q7: What level of committment do you think he has to this issue? Moderate Is Selected Or Q7: What level of committment do you think he has to this issue? Neutral Is Selected Or Q7: What level of committment do you think he has to this issue? Slight Is Selected Or Q7: What level of committment do you think he has to this issue? None Is Selected

Q3.13 Q8: Do you think that other people would be influenced by his support of the issue?

- Yes (1)
- No (2)
- Unsure (3)

Q3.14 Q9: Why do you believe he is appearing in this campaign?Please tick all that apply

- He is knowledgeable about it (1)
- He wants to support it publically (7)
- He is involved in an organisation or charity that is working on this (2)
- He hopes to improve his public profile (3)
- He cares about it (4)
- He is being paid to appear (5)
- A friend asked him to get involved (8)
- Other (please specify) (6) ____________________

Answer If Q9: Why do you believe he is appearing in this campaign? Please tick all that apply q://QID149/SelectedChoicesCount Is Greater Than or Equal to 1

Q3.15 Q10: Can you remember his name? If so, please write it here:

Answer If Q9: Why do you believe he is appearing in this campaign? Please tick all that apply q://QID149/SelectedChoicesCount Is Greater Than or Equal to 1

Q3.16 Q11: Can you remember what the campaign is about? If so, please write it here:

Q4.1 Thank you for agreeing to take part in this short survey - it should only take about 5 minutes to complete.     You are about to see a promotional image for a campaign to stop wildlife poaching. Please take a moment to look at it carefully.

Q4.2

Q4.3 Q1: If you saw this image online, would you click on the link to find out more?

- Yes (1)
- No (2)
- Unsure (3)

Answer If Q1: If you saw this image online, would you click on the link to find out more? Yes Is Selected Or Q1: If you saw this image online, would you click on the link to find out more? No Is Selected Or Q1: If you saw this image online, would you click on the link to find out more? Unsure Is Selected

Q4.4 Q2: Why is that?Please indicate where on the spectrum your opinion lies:

|  | 1 (1) | 2 (2) | 3 (3) | 8 (8) | 10 (10) |
| --- | --- | --- | --- | --- | --- |
| I like him:I don't like him (1) |  |  |  |  |  |
| I am interested in this issue:I am not interested in this issue (2) |  |  |  |  |  |
| I believe he is knowledgeable about it:I don't believe he knows enough about it (3) |  |  |  |  |  |
| His statement caught my attention:His statement has no impact on me (4) |  |  |  |  |  |
| I am interested in why he is supporting it:I don't care that he is supporting it (5) |  |  |  |  |  |
| I often click on promotional images like this:I never click on promotional images like this (6) |  |  |  |  |  |
| I instantly recognised him:I don't know who he is (7) |  |  |  |  |  |

Answer If Q1: If you saw this image online, would you click on the link to find out more? Yes Is Selected Or Q1: If you saw this image online, would you click on the link to find out more? No Is Selected Or Q1: If you saw this image online, would you click on the link to find out more? Unsure Is Selected

Q4.5 Q3: Are there any other reasons for your decision?

Q4.6

Q4.7 Q4: Do you know who he is?

- Yes (1)
- No (2)
- Unsure (3)

Answer If Q4: Do you know who he is? Yes Is Selected

Q4.8 Q5: Please tell us who you think he is and what he is known for:

Answer If Q4: Do you know who he is? No Is Selected Or Q4: Do you know who he is? Unsure Is Selected

Q4.9 His Royal Highness The Duke of Cambridge founded United for Wildlife to bring together the world&#39;s seven leading wildlife organisations to create a global movement for change and raise awareness about the illegal trade in wildlife.     Q5: Knowing this information, does his appearance in this campaign make more sense?

- Yes (1)
- No (2)
- Unsure (3)

Answer If Q5: Please tell us who you think he is and what he is known for: Text Response Is Not Empty Or Q5: Please tell us who you think he is and what he is known for: Text Response Is Empty Or His Royal Highness The Duke of Cambridge founded United for Wildlife to bring together the worl... Yes Is Selected Or His Royal Highness The Duke of Cambridge founded United for Wildlife to bring together the worl... No Is Selected Or His Royal Highness The Duke of Cambridge founded United for Wildlife to bring together the worl... Unsure Is Selected

Q4.10 Q6: Were you aware of his support for this issue prior to seeing this promotional image?

- Yes (1)
- No (2)
- Unsure (3)

Answer If Were you aware of his support for this issue prior to seeing this promotional image? Yes Is Selected

Q4.11 Q7: Does it increase your belief in his commitment to this issue?

- Yes (1)
- No (2)
- Unsure (3)

Answer If Were you aware of his support for this issue prior to seeing this promotional image? No Is Selected Or Were you aware of his support for this issue prior to seeing this promotional image? Unsure Is Selected

Q4.12 Q7: What level of committment do you think he has to this issue?

- Strong (1)
- Moderate (2)
- Neutral (3)
- Slight (4)
- None (5)

Answer If Does his continuing support increase your belief in his committment to this issue? Yes Is Selected Or Does his continuing support increase your belief in his committment to this issue? No Is Selected Or Does his continuing support increase your belief in his committment to this issue? Unsure Is Selected Or What level of committment do you think he has to this issue? Strong Is Selected Or What level of committment do you think he has to this issue? Moderate Is Selected Or What level of committment do you think he has to this issue? Neutral Is Selected Or What level of committment do you think he has to this issue? Slight Is Selected Or What level of committment do you think he has to this issue? None Is Selected

Q4.13 Q8: Do you think that other people would be influenced by his support of the issue?

- Yes (1)
- No (2)
- Unsure (4)

Q4.14 Q9: Why do you believe he is appearing in this campaign?Please tick all that apply

- He is knowledgeable about it (1)
- He wants to support it publically (7)
- He is involved in an organisation or charity that is working on this (2)
- He hopes to improve his public profile (3)
- He cares about it (4)
- He is being paid to appear (5)
- A friend asked him to get involved (8)
- Other (please specify) (6) ____________________

Answer If Why do you believe he is appearing in this campaign? Please tick all that apply q://QID95/SelectedChoicesCount Is Greater Than or Equal to 1

Q4.15 Q10: Can you remember his name? If so, please write it here:

Answer If Q9: Why do you believe he is appearing in this campaign?Please tick all that apply q://QID95/SelectedChoicesCount Is Greater Than or Equal to 1

Q4.16 Q11: Can you remember what the campaign is about? If so, please write it here:

Q5.1 You are about to see some other people who might appear in the promotional campaign. Please take a moment to look at each one.Click "Next" to display the images

Q5.2

Q5.3 Q12: For the four images, please indicate which of the following statements you think applies to each person:Please tick all that apply

|  |  | | | |
| --- | --- | --- | --- | --- |
|  | Crawford Allen (1) | David Beckham (2) | Chris Packham (3) | Duke of Cambridge (4) |
| I believe he is knowledgeable about this issue (1) |  |  |  |  |
| I believe he cares about this issue (2) |  |  |  |  |
| I already knew about his support for this issue (3) |  |  |  |  |
| His statement made me want to find out more (4) |  |  |  |  |
| His photograph caught my eye (5) |  |  |  |  |
| I believe he is using this issue to improve or raise his own profile (6) |  |  |  |  |
| I believe he might be being paid to promote this issue (7) |  |  |  |  |

Q5.4 Q13: Which one would be most likely to make you click on the link in the red box?

- Crawford Allen   (5)
- David Beckham   (6)
- Chris Packham   (7)
- The Duke of Cambridge   (8)

Answer If Which of the four adverts do you prefer? Crawford Allen Is Selected Or Which of the four adverts do you prefer? David Beckham Is Selected Or Which of the four adverts do you prefer? Chris Packham Is Selected Or Which of the four adverts do you prefer? The Duke of Cambridge Is Selected

Q5.5 Q14: Why do you prefer this one?Please tick all that apply

- I like him more than the others (1)
- I already knew about his support for this (2)
- His statement caught my attention (3)
- His photo caught my eye (4)
- I believe he is the most knowledgeable about this issue (6)
- I don't know who the others are (7)
- I was unaware the others supported this issue (10)
- Other, please specify: (8) ____________________

Q6.1 Thank you so much.The survey is nearly complete, all we need now is a little information about you. This information is completely anonymous and can not be used to identify you in any way.

Q6.2 Q15: Please tell us your gender:

- Male (1)
- Female (2)
- Prefer not to say (3)

Q6.3 Q16: Please tell us your age:

- Less than 16 (1)
- 16 to 24 (2)
- 25 to 34 (3)
- 35 to 44 (4)
- 45 to 54 (5)
- 55 to 64 (6)
- 65 to 74 (7)
- 75 to 84 (8)
- More than 85 (9)
- Prefer not to say (10)

Q6.4 Q17: Which of the following best describes where you currently live?

- Hamlet or Isolated Dwelling (2)
- Village (3)
- Town (4)
- City (5)
- Major City (6)
- Prefer not to say (14)

Q6.5 Q18: Have you completed a university degree?

- Yes (1)
- No (2)
- Prefer not to say (3)

Q6.6 Q19: Are you currently:

- Employed (1)
- Self-employed (2)
- Out of work and looking for work (3)
- A homemaker (5)
- A student (6)
- Retired (7)
- Unable to work (8)
- Prefer not to say (9)

Answer If Q19: Are you currently: Employed Is Selected

Q6.7 Q19a: Please describe your employer:

- A for-profit business or individual (1)
- A non-profit organisation, charity or individual (2)
- Local government (3)
- National government (4)
- Self-employed (5)
- Family business or farm (6)
- Prefer not to say (7)

Q6.8 Q20: In the last year have you made any donations to, paid a membership fee towards, or volunteered for any of the following types of organisations?

- Animals - including conservation, welfare and zoos and aquariums (1)
- Arts & Culture - including museums, art galleries, performing arts and public broadcasting (6)
- Education - including scholarships and support (5)
- Environmental - including parks and botanical gardens (2)
- Health - including disease and disorder, and research, treatment and support (4)
- Human Services - including children's, social, homeless and crisis services (8)
- International - including development, disaster relief and human rights (3)
- Religious (7)
- Prefer not to say (9)

Answer If In the last year have you made any donations to, paid a membership fee towards, or volunteered fo... Animals - including conservation, welfare and zoos and aquariums Is Selected Or In the last year have you made any donations to, paid a membership fee towards, or volunteered fo... Environmental - including parks and botanical gardens Is Selected

Q6.9 Q21: Which of these organisations do you support, either through donations, membership or as a volunteer:Please tick all that apply.

- BES - British Ecological Society (2)
- Conservation International (10)
- Durrell Wildlife Conservation Trust (8)
- Fauna & Flora International (5)
- Greenpeace (14)
- The Nature Conservancy (11)
- RSPB - Royal Society for the Protection of Birds (1)
- WCS - Wildlife Conservation Society (12)
- The Wildlife Trusts (4)
- WWF - World Wide Fund for Nature (3)
- WWT - Wildfowl & Wetlands Trust (19)
- ZSL - London Zoo / Whipsnade Zoo (7)
- Other, please specify: (6) ____________________
- Prefer not to say (18)

Q6.10 Finally, if you would like to make any additional comments about anything you have been asked in this survey, please use the space below:
