# Supplementary material for: The effectiveness of celebrities in conservation marketing
Source: PLoS One. 2017 Jul 7;12(7):e0180027. doi: 10.1371/journal.pone.0180027 (PMC5501471; doi:10.1371/journal.pone.0180027)
Supplement: S4 Appendix — (DOCX) [file pone.0180027.s004.docx]

***Appendix S4: Predictors used in models of respondents’ willingness-to-engage with (W) and recall (R) of adverts presented singly and choices (C) between four alternative adverts presented side by side.***

| Variable(s) | Question | Modelled as | Levels / Further details | Analysis | Alternative-/Individual-specific | N. missing |
| --- | --- | --- | --- | --- | --- | --- |
| treatment | Advert | Categorical | - David Beckham - Chris Packham - Prince William - Crawford Allen | W + R | -- | 0 |
| why_clicked_X | Q2 | Multiple continuous | (See questionnaire for specific 5 point Likert-type items) | W + R | -- | Like: 42  Interested: 27  Knowledge: 43  Attention: 29  Interest why: 43  Often click: 32  Recognised: 31 |
| know_who | Q4 | Categorical | - Yes - No - Unsure | W + R | -- | 3 |
| aware | Q6 | Categorical | - Yes - No - Unsure | W + R | -- | 8 |
| other_influence | Q8 | Categorical | - Yes - No - Unsure | W + R | -- | 32 |
| why_appearing_X | Q9 | Multiple binary | (See questionnaire for specific statements) | W + R | -- | 0 |
| initially_shown | Advert | Categorical | - David Beckham - Chris Packham - Prince William - Crawford Allen | C | I | 0 |
| photo; statement; care; knowledge; knew; paid; profile | Q12 | Multiple categorical (See questionnaire for specific statements) | - David Beckham - Chris Packham - Prince William - Crawford Allen | C | A | 0 |
| why_prefer_X | Q14 | Multiple binary | (See questionnaire for specific statements) | C | I | 0 |
| gender | Q15 | Categorical | - Male - Female - Prefer not to say | W + R + C | I | 0 |
| age_c | Q16 | Continuous | - 1 = 16 – 24 - 2 = 25 – 34 - 3 = 35 – 44 - 4 = 45 – 54 - 5 = 55 – 64 - 6 = 65+ | W + R + C | I | 0 |
| urban_scale | Q17 | Continuous | - 1 = Countryside - 2 = Village - 3 = Town - 4 = City - 5 = Major city | W + R + C | I | 0 |
| education | Q18 | Categorical | - Yes - No - Prefer not to say | W + R + C | I | 0 |
| employment | Q19 | Categorical | - Employed - Self-employed - Student - Other | W + R + C | I | 0 |
| charity_X | Q20 | Multiple binary | (See questionnaire for specific charities) | W + R + C | I | 0 |
| organisations | Q21 | Binary | - 0 = None - 1 = One or more supported | W + R + C | I | 0 |
